# Medical College Commencements

**Published:** 1893-03

**Authors:** 


### MEDICAL COLLEGE COMMENCEMENTS.

The thirty-fifth annual commencement of the Atlanta Medical College was held at DeGive's opera house the evening of March 1st.

Rev. H. C. Morrison, D. D., opened the exercises with prayer.

The Proctor, Dr. W. S. Kendrick, made his annual report, in which he stated that there had been nearly two hundred students in attendance during the session just closed. He stated that the college building had been made one of the best in the country, at great expense, being provided with steam heat, thorough water supply and sewerage; and complete chemical apparatus and microscopes imported directly from Europe.

The pharmaceutical department closed its second session under the most favorable auspices.

He pledged the cordial support of the college and faculty to the pending law requiring higher medical education.

Nine States were represented by the students in the medical department, and three in the pharmaceutical department.

The Hon. N. J. Hammond, President of the Board of Trustees, then conferred the degrees upon seventy-six graduates in medicine and four graduates in pharmacy.

Hon. Ben. J. Conyers, one of the most brilliant men in the State, delivered the oration of the evening, a most eloquent and scholarly address.

Dr. J. M. Thomas, of Atlanta, delivered an excellent valedictory address, making a special plea that the college establish a a school for the training of nurses.

Judge W. R. Hammond then conferred the "honor medals," as follows:

First honor—Dr. William Wightman Mangum, of Alabama.

Second honor—Dr. James LeRoy Campbell, of Georgia.

Third honor—Dr. N. Wilson Gable, of Georgia.

Those to whom honorable mention was given were: Drs.

W. H. Bryan, J. E. Murray, W. D. Hicks, G. L. Alexander, J. C. Mull and J. C. Hooten.

---

THE Southern Medical College held its fourteenth annual commencement in DeGive's opera house on the night of the 2d instant. The Dean of the college, Dr. Nicolson, made his report to the Board of Trustees, in which he stated that the college was now enjoying an unprecedented prosperity; within the last year a new college building had been erected, especially adapted for the purpose of medical teaching. The Dean took pleasure in reporting that the college had united with the Southern Medical College Association, and that it was in hearty sympathy with the demand for better medical education.

The graduating class numbered thirty-seven. The valedictorian was Dr. John R. Shannon, of Georgia. The distinction of first honor was shared by three gentlemen, Drs. J. McF. Gaston, Jr., of Atlanta, D. H. Perry, of Florida, and F. L. Dennis, of Atlanta. The second honor was awarded to Dr. W. C. Hathcock, of Atlanta; the third to Dr. George R. Plummer, of Florida.

---

DURING the whole of the late Mr. Blaine's long sickness the anxious public were never informed as to the exact nature of his malady. Since his death his attending physicians have made an official statement from which it appears that he was a victim of chronic Bright's disease, as has been often suspected and suggested. His earliest signs of ill health were said to be due to a gouty tendency associated with anemia and progressive malnutrition. It is probable that kidney lesions have been present for many years, and no doubt his attack of paralysis in 1887 was due to an associated endarteritis of the blood vessels of the brain. During the past summer the diagnosis of a progressive nephritis was established beyond doubt. For the last two months of his life he suffered greatly with lung complications, and tubercle bacilli were found in his sputa. In December cardiac weakness set in, which was more or less constant up to the end. As a result of the latter œdema of the lungs occurred, and he died January 27th.

